# Supplementary figures and images for: Antibody Binding and Neutralizing Targets within the Predicted Structure of the Poxvirus Multiprotein Entry-Fusion Complex
Source: bioRxiv. 2025 May 7:2025.05.07.652617. Preprint. [Version 1] doi: 10.1101/2025.05.07.652617 (PMC12190349; doi:10.1101/2025.05.07.652617)

Fig. S1

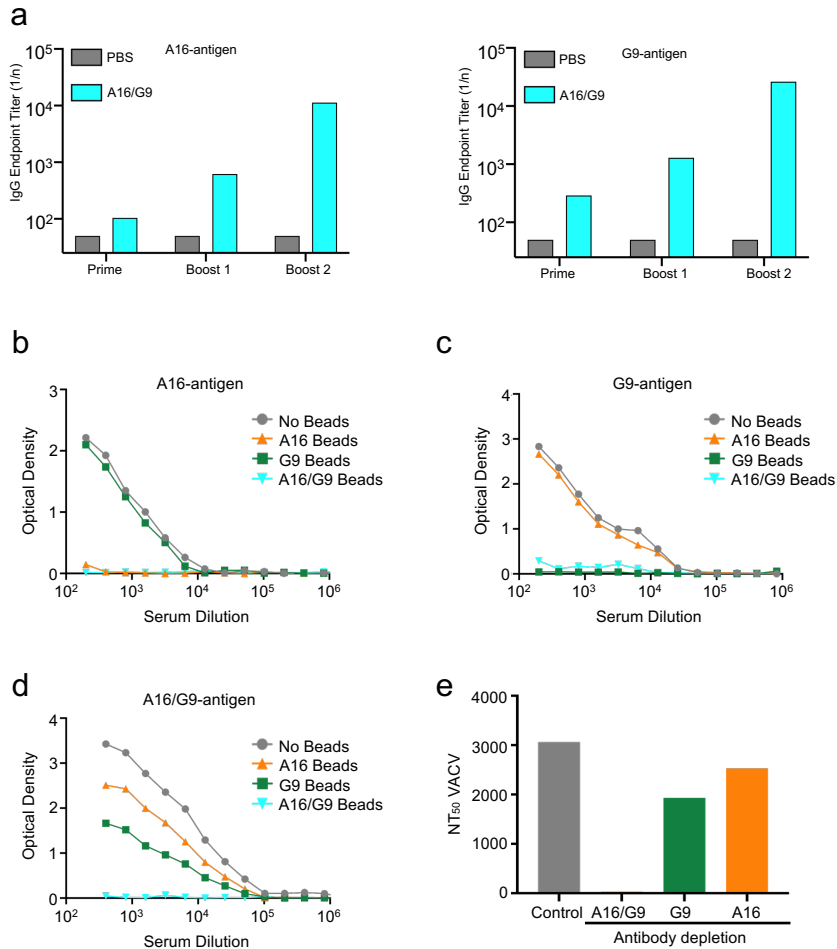

Fig. S2

a

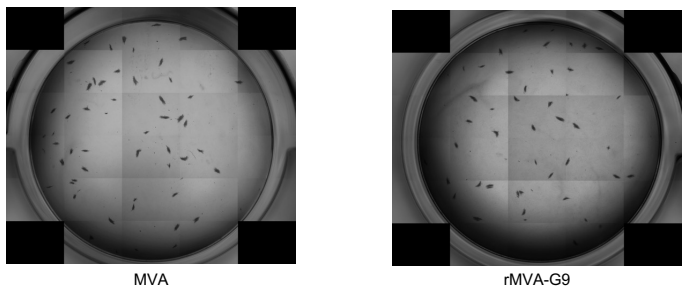

b

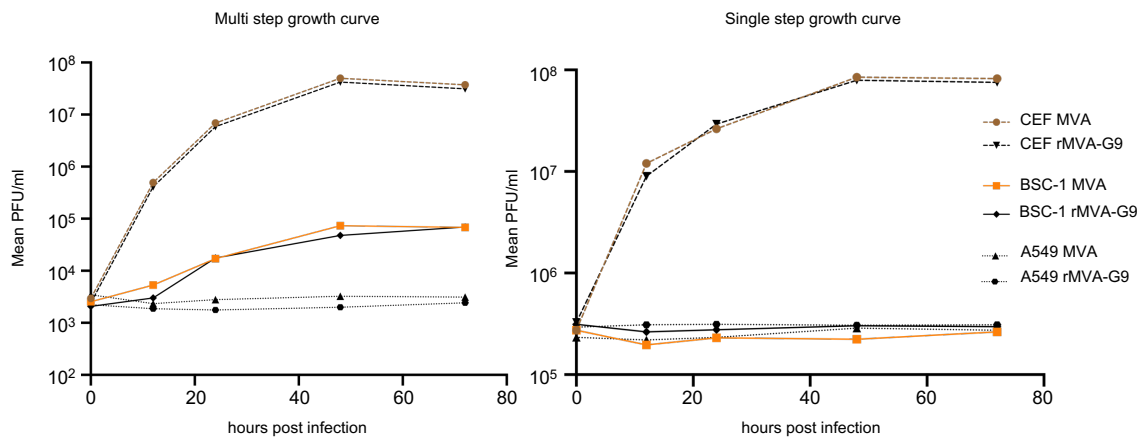

a

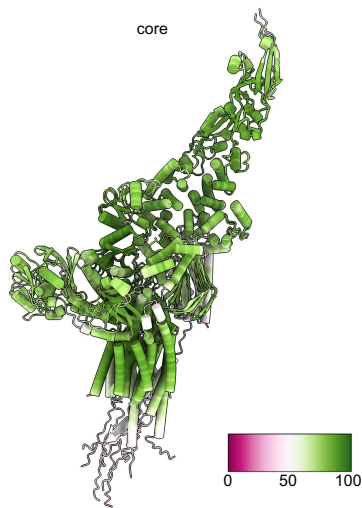

c

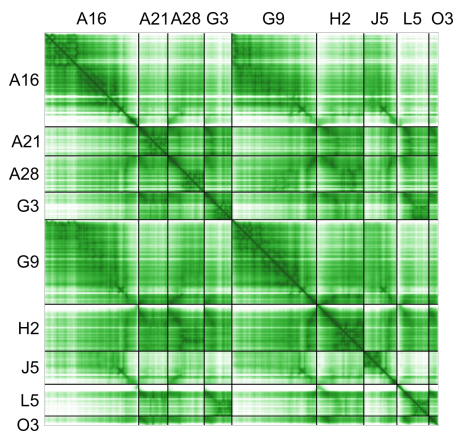

b

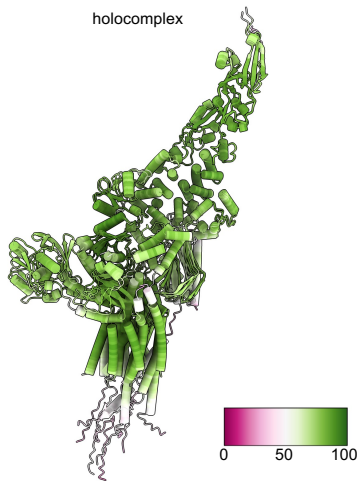

d

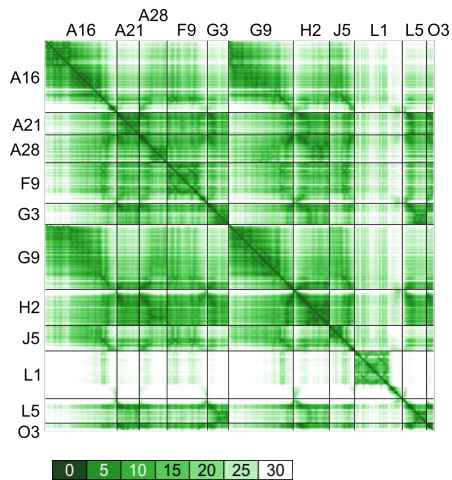

Fig. S4

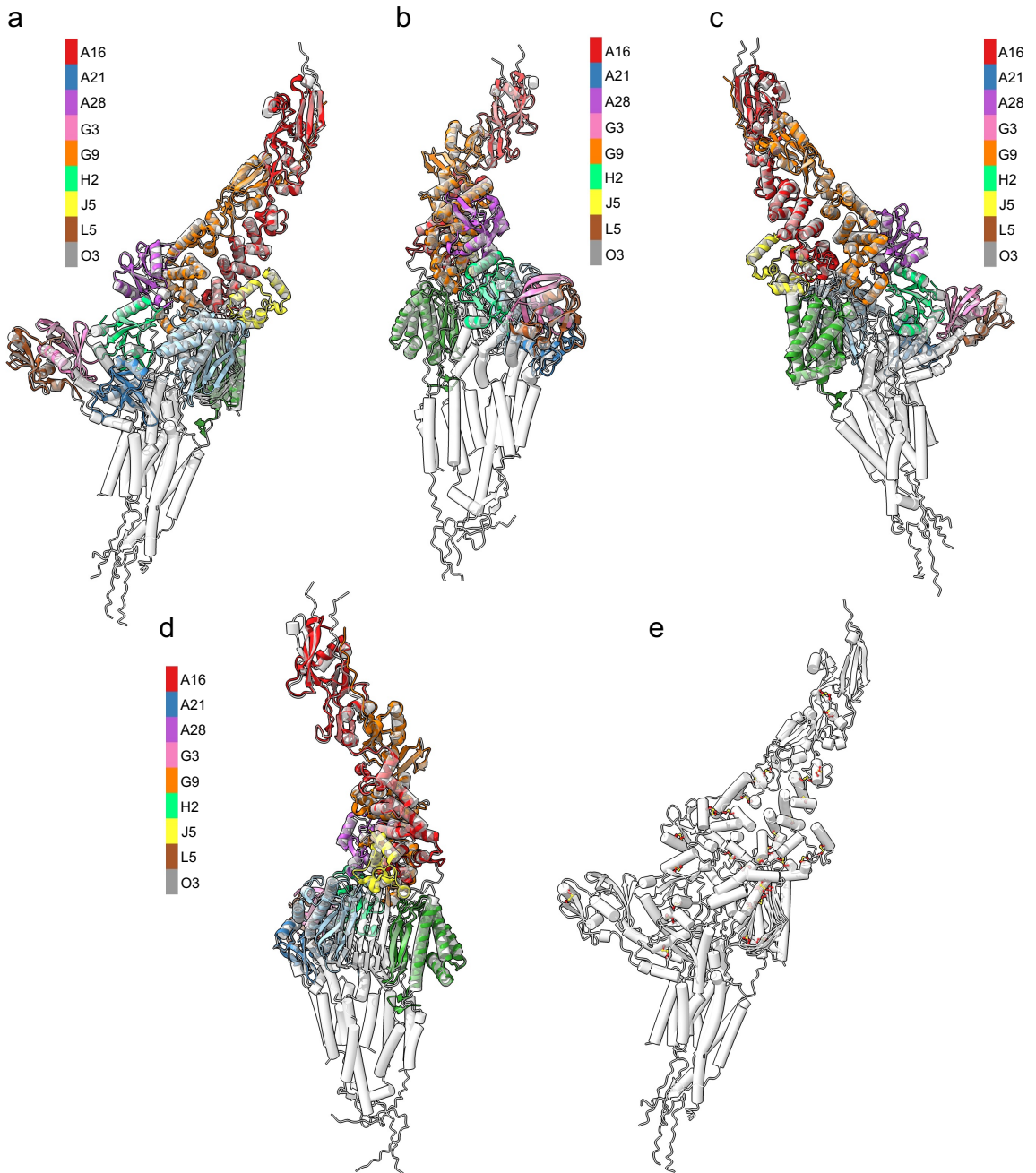

Fig. S5

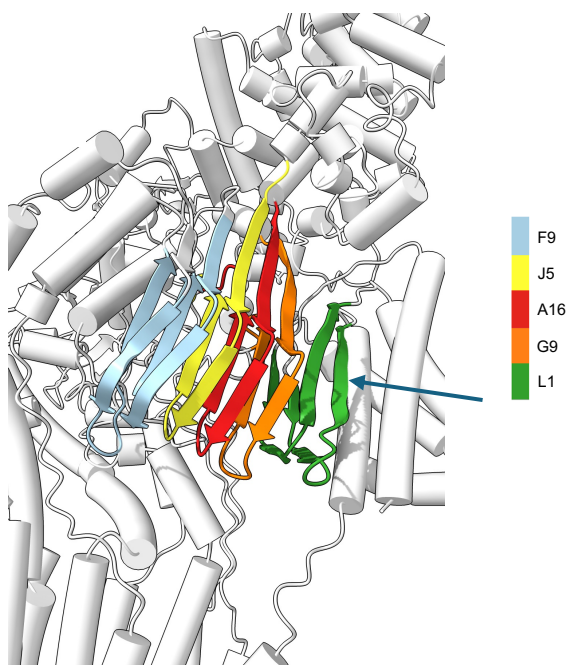

Supplement: 1 — Fig. S1. Binding and depletion of antibodies from mice immunized with A16/G9. (a) Binding of A16/G9 serum to A16 and G9 antigens. (b-d) Depletion of A16, G9 and A16/G9 by proteins bound to NiNTA beads determined by ELISA. (e) Depletion of VACV neutralizing activity in serum of mice immunized with A16/G9 heterodimers. Fig. S2. Replication of recombinant MVA expressing secreted G9. (a) Virus foci formed in CEF monolayers within 48 h and stained with anti-VACV antibodies. (b) Multistep (0.1 PFU) and single step (10 PFU) growth curves of MVA and recombinant MVA following infection of BS-C-1, CEF and A549 cells. Fig. S3. Confidence metrics of predicted core and holocomplex EFC models. The predicted local difference test (pLDDT) per-residue measure of local confidence of core (a) and holocomplex (b) models scaled 0 to 100 with higher score indicating higher confidence. Predicted aligned error (PAE) measure of confidence in relative position of two residues within predicted core (c) and holocomplex (d) models. Regions of high confidence are dark green. Fig. S4. Correlation of predicted and experimental structures. (a-d) The predicted holocomplex in light gray with superimposed ectodomain structures from the Protein Database (PDI) is shown in four 90° rotations. Individual proteins identified by color. (e) Close association of cysteine residues in holocomplex model. Pairs of cysteines in red. Fig. S5. Model of holocomplex showing alignment of β-folds of A16, G9, J5 and F9. β-folds in color. [file NIHPP2025.05.07.652617V1-supplement-1.pdf]
